# Articulatory undershoot of vowels in isolated REM sleep behavior disorder and early Parkinson’s disease

**DOI:** 10.1038/s41531-022-00407-7

**Published:** 2022-10-20

**Authors:** Dominik Skrabal, Jan Rusz, Michal Novotny, Karel Sonka, Evzen Ruzicka, Petr Dusek, Tereza Tykalova

**Affiliations:** 1 Department of Neurology and Centre of Clinical Neuroscience, First Faculty of Medicine, Charles University and General University Hospital, Prague, Czech Republic; 2 Department of Circuit Theory, Faculty of Electrical Engineering, Czech Technical University in Prague, Prague, Czech Republic; 3 Department of Neurology & ARTORG Center, Inselspital, Bern University Hospital, University of Bern, Bern, Switzerland

**Keywords:** Parkinson's disease, Olfactory system

## Abstract

Imprecise vowels represent a common deficit associated with hypokinetic dysarthria resulting from a reduced articulatory range of motion in Parkinson’s disease (PD). It is not yet unknown whether the vowel articulation impairment is already evident in the prodromal stages of synucleinopathy. We aimed to assess whether vowel articulation abnormalities are present in isolated rapid eye movement sleep behaviour disorder (iRBD) and early-stage PD. A total of 180 male participants, including 60 iRBD, 60 de-novo PD and 60 age-matched healthy controls performed reading of a standardized passage. The first and second formant frequencies of the corner vowels /a/, /i/, and /u/ extracted from predefined words, were utilized to construct articulatory-acoustic measures of Vowel Space Area (VSA) and Vowel Articulation Index (VAI). Compared to controls, VSA was smaller in both iRBD (*p* = 0.01) and PD (*p* = 0.001) while VAI was lower only in PD (*p* = 0.002). iRBD subgroup with abnormal olfactory function had smaller VSA compared to iRBD subgroup with preserved olfactory function (*p* = 0.02). In PD patients, the extent of bradykinesia and rigidity correlated with VSA (*r* = −0.33, *p* = 0.01), while no correlation between axial gait symptoms or tremor and vowel articulation was detected. Vowel articulation impairment represents an early prodromal symptom in the disease process of synucleinopathy. Acoustic assessment of vowel articulation may provide a surrogate marker of synucleinopathy in scenarios where a single robust feature to monitor the dysarthria progression is needed.

## Introduction

Isolated rapid eye movement sleep disorder (iRBD) is a parasomnia characterized by dream-enactment behavior and loss of physiologic muscle atonia during the rapid eye movement sleep phase. iRBD is considered a prodromal stage of neurodegeneration as more than 80% of diagnosed patients developed alfa-synuclein-aggregation disorders such as Parkinson’s disease (PD), Lewy body dementia, or multiple system atrophy^1,2^. Considering the development of Parkinson’s disease-modifying treatment^3^, a multicentre study including 1280 iRBD patients identified quantitative fine motor skill testing as the strongest predictor for conversion^4^. Another study by Postuma et al. ^5^ revealed that voice and face akinesia represent the earliest prodromal motor manifestations in iRBD subjects preceding the onset of parkinsonism by a mean 9.8 years. This is likely a consequence of motor speech complexity and its sensitiveness to neural damage^6^.

Hypokinetic dysarthria of PD, which is mainly characterised by articulatory, phonatory and prosodic alterations, occurs in up to 90% of patients over the course of the disease^6,7^. Moreover, speech impairment is present in a majority of newly diagnosed PD patients^8,9^. Considering that patients with iRBD are at high risk of developing PD, the speech behavior assessment in iRBD is subjected to thorough investigation. Recent multilanguage research based on fully automated analysis of seven distinctive speech dimensions of hypokinetic dysarthria^10^, including harsh voice, slow sequential motion rates, imprecise consonants, monoloudness, monopitch, prolonged pauses, and articulation rate performed on 150 iRBD patients revealed that only monopitch was able to significantly differentiate iRBD patients from controls^11^. Interestingly, monopitch was found in iRBD subjects with impaired olfactory function before the nigrostriatal dopaminergic transmission is affected^12^, that is, in Braak stage 2 before the substantia nigra is affected by synucleinopathy^13^. Among monopitch, vowel articulation impairment represents one of the core deficits contributing to dysarthric speech, as it reflects the range of articulatory movements and strongly correlates with overall intelligibility^14–16^. The potential of imprecise vowel articulation to serve as an early biomarker can also be supported by a previous pilot study where deficits in vowel articulation were detected in a small sample of 20 patients with de novo PD^17^. However, potential changes of vowel articulation in iRBD have never been investigated. Also, no previous research independently related articulation impairment to other essential prodromal features of synucleinopathy, such as olfactory dysfunction.

The purpose of this study is to investigate vowel articulation in iRBD and early-stage PD patients compared to healthy controls in order (i) to verify the prospect of using measurement of vowel articulation as a biomarker for the detection of prodromal PD and (ii) to investigate the relationship between articulation measures and the degree of motor and smell dysfunction.

## Results

### Group differences

**Fig. 1:**
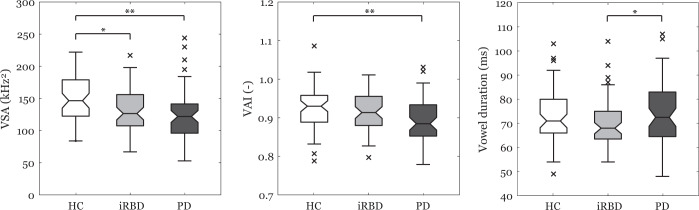
Comparison of vowel measurements including VSA, VAI and vowel duration between HC, iRBD and PD using boxplots. The centre line indicates the median, and the bounds of the box indicate the 25th and 75th percentiles. The whiskers extend to the most extreme data points not considered outliers, and the outliers are plotted individually using the ‘x’ symbol. VSA, vowel space area, VAI vowel articulation index, “Asterisks” indicate significant differences after Bonferroni correction: *p < 0.05; **p < 0.01.

**Fig. 2:**
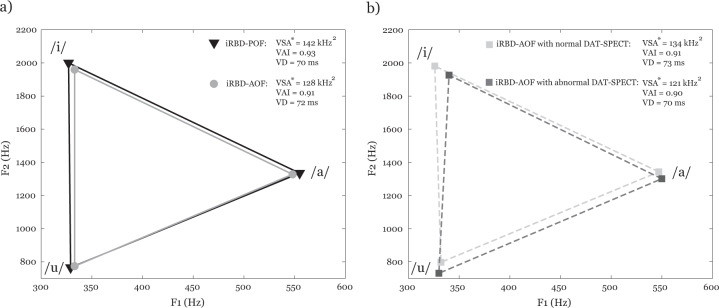
Mean F1 and F2 values and vowel space area for a) iRBD-POF compared iRBD-AOF subgroups and b) iRBD-AOF with normal DAT-SPECT compared to iRBD-AOF with abnormal DAT-SPECT. VSA vowel space area, VAI vowel articulation index, VD vowel duration, iRBD-POF isolated rapid eye movement sleep behaviour disorder patients with preserved olfactory function, iRBD-AOF isolated rapid eye movement sleep behaviour disorder patients with abnormal olfactory function, DAT-SPECT dopamine transporter single-photon emission computed tomography; “Asterisks” indicate significant differences: *p < 0.05.

Normative values of the first two formants for iRBD, PD and healthy control (HC) groups are summarized in Table 1. Vowel Space Area (VSA) was found to be the best parameter for differentiating between groups [F(2,177) = 7.4, *p* = 0.001, *η*^2^ = 0.08] (Fig. 1). Post hoc comparisons revealed significantly smaller VSA in both iRBD (*p* = 0.01) and PD (*p* = 0.001) compared to HC individuals. In addition, group differences were also detected for Vowel Articulation Index (VAI) [F(2,177) = 6.3, *p* = 0.002, *η2* = 0.07], as the PD group manifested significantly smaller VAI (*p* = 0.002) compared to HC group. Slight vowel duration differentiation was also observed across groups [F(2,177) = 3.2, *p* = 0.04, *η*^2^ = 0.04), associated with differences between PD and iRBD groups (*p* = 0.04). The sub-experiment concerning olfactory function in iRBD showed that iRBD group with preserved olfactory function (iRBD-POF) had greater VSA than iRBD group with abnormal olfactory function (iRBD-AOF) [F(1,54) = 5.4, *p* = 0.024, *η*^*2*^ = 0.094] (Fig. 2a). In addition, iRBD-AOF with normal dopamine transporter single-photon emission computed tomography (DAT-SPECT) showed greater VSA than iRBD-AOF with abnormal DAT-SPECT [F(1,31) = 4.2, *p* = 0.049, *η*^*2*^ = 0.140] (Fig. 2b). No significant differences for VAI and vowel duration were found.

**Table 1:** Normative values of formant frequencies in male groups of iRBD, PD and HC.

	iRBD	PD	HC
	mean/SD (range)	mean/SD (range)	mean/SD (range)
F1/a/ (Hz)	548/43 (448−627)	548/53 (452−676)	582/49 (497−667)
F2/a/ (Hz)	1331/78 (448−627)	1328/81 (1172−1567)	1350/86 (1119−1535)
F1/i/ (Hz)	331/16 (300−376)	332/18 (293−380)	348/23 (304−408)
F2/i/ (Hz)	1980/108 (1680−2179)	1931/128 (1703−2190)	2012/103 (1750−2226)
F1/u/ (Hz)	331/19 (294−377)	337/20 (295/401)	340/20 (299−405)
F2/u/ (Hz)	770/64 (661−957)	786/52 (680−894)	762.9/54 (666−903)

*iRBD*, isolated rapid eye movement sleep behaviour disorder, *PD* Parkinson’s disease, *HC* Healthy control, *F1* First formant frequency, *F2* Second formant frequency

### Correlations between speech and motor variables

**Fig. 3:**
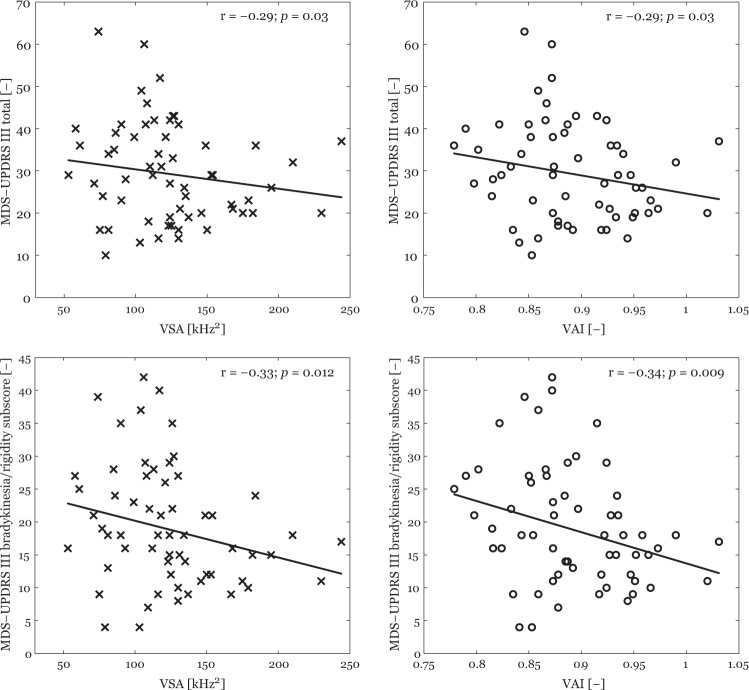
Significant correlations between clinical motor scales and acoustic data plotted to 2D space with the trend of averaged data (black line) in the PD group. VSA Vowel space area, VAI Vowel articulation index, MDS–UPDRS III Movement Disorder Society – Unified Parkinson’s Disease Rating Scale part III.

Movement Disorder Society Unified Parkinson’s Disease Rating Scale motor part (MDS-UPDRS III) total in PD patients showed negative correlation with VSA (*r* = −0.29, *p* = 0.03) and VAI (*r* = −0.29, *p* = 0.03). In addition, bradykinesia and rigidity subscore in PD patients showed negative correlation with VSA (*r* = −0.33, *p* = 0.01) and VAI (*r* = −0.34, *p* < 0.01) while neither correlation between postural instability and gait difficulty (PIGD) subscore and VSA (*r* = −0.04, *p* = 0.75) or VAI (*r* = −0.12, *p* = 0.75) nor between tremor subscore and VSA (*r* = 0.01, *p* = 0.96) or VAI (*r* = 0.06, *p* = 0.64) was detected (Fig. 3).

Regarding brain imaging, the putamen binding ratio in iRBD showed positive correlation with VSA (*r* = 0.35, *p* = 0.01). No other significant correlations were found between vowel articulation parameters and clinical scales in PD or iRBD.

## Discussion

Results of our study revealed that subtle impairment in vowel articulation due to the reduced articulatory range of motion is already evident in prodromal synucleinopathy. Articulatory impairment in iRBD was detectable through objective acoustic analysis despite almost no perceptual dysarthria severity was noted during clinical examination. Since the extent of articulatory undershoot was related to bradykinesia and rigidity in our PD cohort, we may hypothesize that vowel articulation abnormalities in parkinsonism result mainly as a consequence of nigrostriatal degeneration. The strength of this study is that vowel articulation features were evaluated in a large sample of iRBD and de-novo PD patients. Examining drug-naïve patients is especially important as dopaminergic treatment may improve certain aspects of speech disorder such as vowel articulation^18^. Since the vowel articulation performance of iRBD subjects intermediated between controls and de-novo PD patients and since it was more severe in iRBD with hyposmia compared to iRBD with preserved smell function, acoustic assessment of vowels could be potentially useful as a diagnostic and prognostic biomarker in α-synuclein-aggregation disorders.

Our findings of lowered vowel space in iRBD demonstrate that vowel articulation is already altered in the prodromal stages of synucleinopathy. In particular, vowel articulation was more impaired in iRBD subgroup with severe hyposmia, which is one of the most common and earliest non-motor prodromal features to emerge in PD^2,4^. Thus, we might assume that vowel articulation impairment is an early prodromal symptom that progresses along with olfactory dysfunction in the disease process of synucleinopathy. Accordingly, recent research demonstrated that dysprosody is already present in iRBD subjects with impaired olfactory function but still intact nigrostriatal pathway^12^. Together, these findings might indicate that speech production is already slightly affected in Braak’s stage 2, which is associated with Lewy pathology within brainstem nuclei^13^, a brain region crucial for controlling vocal fold tension^19^.

Furthermore, our findings in iRBD subgroup with impaired olfactory function implies that articulatory undershoot is also result of nigrostriatal neurodegeneration, as greater vowel deficits were found in the subgroup of patients with abnormal compared to those with normal DAT-SPECT. This assumption can be further supported by the observed link between the extent of vowel articulation decline and bradykinesia and rigidity but not axial gait symptoms in our de-novo PD group. Also, the previous pilot study discovered a positive correlation between amelioration of vowel articulation and dopaminergic treatment-related improvement in bradykinesia and rigidity^18^. On the other hand, degeneration in non-dopaminergic brain regions may further contribute to the worsening of vowel articulation performance as PD progresses. This is in agreement with a previous longitudinal study reporting a further decline of vowel articulation in the course of the disease in PD patients with an average disease duration of 6 years after the diagnosis^20^. Indeed, it is well known that vowel articulation impairments strongly correlate with overall intelligibility^14–16^, which tends to be affected in the later stages of PD. A recent study showed that articulatory deficits in de-novo PD patients are indicative of more widespread brain damage affecting extranigral cortical or subcortical regions^8^. In accordance with this assumption, a former study reported that aggravation of dysarthria during PD progression results particularly from the increasing severity of cerebral non-dopaminergic lesions^21^. Finally, in PD patients treated with bilateral subthalamic nucleus deep brain stimulation, the severity of the residual parkinsonian speech score was predictive of a poor postoperative outcome, likely due to the presence of non-dopaminergic lesions within the brain^22^. However, we cannot exclude that vowel articulation impairment in advanced PD may be also related at least in part to levodopa-induced dyskinesia^23^, together with the neurodegeneration of dopaminergic and non-dopaminergic brain areas. In summary, given the existing evidence in literature, we may hypothesize that vowel articulation deficits, along with limb bradykinesia, are primarily related to dopaminergic involvement in the early stages, whereas nondopaminergic lesions further contribute to the worsening of vowel articulation in the later stages of PD. Therefore, acoustic assessment of vowel articulation may provide a surrogate marker of neurodegeneration from prodromal to more advanced synucleinopathy for scenarios where a single robust feature to monitor the dysarthria progression is desired.

In our study, both VSA and VAI reflected speech impairment in drug-naive PD patients, which is in accordance with a previous pilot study investigating a small sample of 20 de-novo PD patients^17^. Contrary to our results on de-novo PD patients, several authors suggested VAI is superior over VSA in moderate to advanced stages of PD^20,24^. The principle of VAI construction focuses on formant centralization in order to minimize the effect of interspeaker variability; as a consequence, VAI may not reflect subtle articulatory spatial modifications^25^. On the other hand, VSA is calculated out of the maximal extent of vowel working space, which might better mirror subtle speech changes^18,26^. In other words, we may hypothesize that PD vowel articulatory impairment is tongue-dominant and emerge initially in the posterior parts of an articulatory organ^26,27^. Since the individual vowel /u/, which is characterised with tongue positioning high and backward^28^, seem to be the most affected vowel in early stages of PD^17^, VSA construction shall significantly acknowledge single-vowel frequency shift, whereas the sensitivity of VAI might be lowered as it accounts for centralization of all corner vowel that does not need to be affected in the early disease process.

Previous studies assumed that speaking rate may influence vowel articulation performance in dysarthrias^29^. However, we did not find differences for vowel duration at the group level between HC and both patient cohorts, likely as a consequence of the very early stages of synucleinopathy investigated. Despite inconclusive results regarding articulation rate in later stages of synucleinopathies, including no changes in the speech rate, decreased speech rate, or even an accelerated speech rate^30–32^, our findings are in agreement with a recent study showing no speech rate changes in de-novo PD and only a trend toward slower speech rate in iRBD^33^. Faster speech observed in advanced PD is likely to reflect a physiological tendency to accelerate speech due to the impaired motor planning (oral festination)^30,31^ whereas the tendency toward a slower speech rate may be theoretically attributed to the degeneration of non-dopaminergic pathways^8^.

This study has certain limitations. We enrolled exclusively male participants primarily because of the strong predominance of male subjects within iRBD patients^34^. Anatomical dispositions of the vocal tract reflect sexual dimorphism^35,36^, and we therefore cannot exclude a potential sex effect on our results. On the other hand, no sex-specific speech dysfunction in de-novo PD was found^9^. The findings of current study are based on a cross-sectional design. Future research is needed to estimate the sensitivity of vowel articulation features in long-term follow-up and in predicting phenoconversion from iRBD to established parkinsonism.

In conclusion, the vowel articulation deficits in male subjects significantly differentiated both iRBD and de-novo PD patients from controls. Future studies should elaborate our findings in the female population, and vowel articulation analysis might then have the potential to serve as a speech biomarker indicating the prodromal stage of synucleinopathy. Introducing novel acoustic biomarkers enable to design a speech assessment battery allowing for valid and easy evaluation of a large number of subjects in clinical trials as speech assessment is inexpensive, noninvasive and digitally storable. Additionally, vowel articulation analysis may facilitate better speech phenotype classification of PD and has, thus, the potential utility for personalized medicine.

## Methods

### Participants

From 2015 to 2021, a total of 180 male Czech participants, including a separate group of iRBD, PD, and HC participants, were recruited. Each participant provided written informed consent. The study received approval from the Ethics Committee of the General University Hospital in Prague, Czech Republic, and has been performed in accordance with the ethical standards established in the 1964 Declaration of Helsinki.

The iRBD group consisted of 60 male patients aged 65.6 (SD 7.1) years diagnosed according to the third edition of the International Classification of Sleep Disorders^37^. None of the iRBD patients suffered a significant communication disorder or had a history of treatment with antiparkinsonian medication, nor were they taking any medication affecting sleep, cognition or motor function. ﻿The PD group consisted of 60 untreated drug-naïve male patients aged 61.8 (SD 11.6) years, fulfilling the Movement Disorder Society clinical diagnostic criteria for PD^38^. At the time of examination, none of the PD patients had a history of communication disorder unrelated to PD, nor were they taking any medication affecting sleep or cognition function.

Both iRBD and PD subjects were examined by a movement disorder specialist (P.D.) using the MDS-UPDRS III^39^. Symptom duration was assigned based on the self-reported occurrence of the first motor symptoms. To define specific movement disorder manifestations that may influence speech disorder, three subscores from the MDS-UPDRS III scale were calculated as follows: (i) PIGD subscore (MDS-UPDRS part III, 3.9 Arising from the chair, 3.10 Gait, 3.11 Freezing of gait, 3.12 Postural stability, 3.13 Posture), (ii) Bradykinesia and Rigidity subscore (MDS-UPDRS part III, 3.3 Rigidity, 3.4 Finger tapping, 3.5 Hand movements, 3.6 Pronation-supination movements of hands, 3.7 Toe-tapping, 3.8 Leg agility, 3.14 Body bradykinesia) and Tremor subscore (MDS-UPDRS part III, 3.15 Postural tremor of the hands, 3.16 Kinetic tremor of the hands, 3.17 Rest tremor amplitude, 3.18 Constancy of rest tremor). Item 3.1 Speech of the MDS-UPDRS III was used for the perceptual description of overall dysarthria severity. In addition, patients were examined using the University of Pennsylvania Smell Identification Test (UPSIT)^40^, Montreal Cognitive Assessment and Scales for Outcomes in Parkinson’s Disease - Autonomic Dysfunction^41^. iRBD patients were categorized into two groups based on their olfactory function: iRBD group with preserved olfactory function (iRBD-POF) consisted of patients with UPSIT > 25 (i.e. normosmic to moderately microsmic) and the iRBD group with abnormal olfactory function (iRBD-AOF) of patients with UPSIT ≤ 25 (i.e. severely microsmic or anosmic). Patient clinical and demographic characteristics are summarised in Table 2.

**Table 2:** Patient clinical and demographic characteristics.

	PD, *n* = 60	iRBD, *n* = 60	iRBD-POF, *n* = 23	iRBD-AOF, *n* = 32
	Mean/SD (range) or *n* (%)	Mean/SD (range) or *n* (%)	Mean/SD (range) or *n* (%)	Mean/SD (range) or *n* (%)
**Clinical characteristics**				
Male gender	60 (100%)	60 (100%)	23 (100%)	32 (100%)
Age (years)	61.8/11.6 (34−81)	65.6/7.1 (46−81)	63.7/8.5 (46−77)	67.6/5.7 (57−81)
Symptom duration (years)	1.1/1.5 (0.3−5.9)	6.5/5.5 (1−28)	5.8/4.0 (1−13)	8.5/8.4 (1−39)
MoCA	25.5/3.1 (17−30)	24.1/2.7 (18−30)	24.5/2.5 (20−29)	23.8/3.0 (18−30)
SCOPA-AUT	8.8/5.2 (0-23)	12.4/7.6 (1-33)	15.3/9.4 (1-33)	11.0/5.6 (2-23)
UPSIT^a^	20.9/7.5 (2-35)	22.1/7.9 (9-37)	30.2/3.4 (26-37)	16.3/4.5 (9-24)
MDS-UPDRS III total	22.5/11.8 (10−63)	6.8/6.2 (0−25)	6.0/5.9 (0−22)	7.3/6.4 (0−25)
MDS-UPDRS III speech item	0.5/0.5 (0−2)	0.1/0.3 (0−1)	0.0/0.0 (0−0)	0.1/0.3 (0−1)
MDS-UPDRS III tremor subscore	6.3/4.0 (0−15)	2.3/2.5 (0−11)	2.3/3.0 (0−11)	2.1/2.4 (0−9)
MDS-UPDRS III PIGD subscore	2.5/1.5 (0−6)	0.7/0.9 (0−4)	0.5/0.7 (0−2)	0.9/1.0 (0−4)
MDS-UPDRS III bradykinesia and rigidity subscore	12.5/9.1 (4−42)	0.6/0.8 (0−3)	2.7/3.1 (0−11)	3.7/4.4 (0−20)
iRBD presence	17 (28%)	60 (100%)	23 (100%)	32 (100%)
**Brain imaging (DAT-SPECT)**				
Caudate binding ratio	2.8/0.6 (1.3−3.8)	3.6/0.7 (2.3−5.4)	3.8/0.7 (2.5−5.3)	3.4/0.6 (2.3−5.4)
Putamen binding ratio	1.5/0.4 (0.8−2.5)	2.8/0.7 (1.3−4.5)	3.1/0.6 (1.9−4.1)	2.6/0.7 (1.3−4.5)
Abnormal DAT-SPECT	60 (100%)	16 (27%)	3 (13%)	12 (38%)

*PD* Parkinson’s disease, *iRBD* isolated rapid eye movement sleep behaviour disorder, *iRBD-POF* iRBD patients with preserved olfactory function, *iRBD-AOF* iRBD patients with abnormal olfactory function, *MDS-UPDRS* Movement Disorder Society Unified Parkinson disease rating scale, *PIGD* Postural instability and gait difficulty, *MoCA* Montreal cognitive assessment, *SCOPA-AUT* Scales for Outcomes in Parkinson’s Disease - Autonomic Dysfunctionm, *UPSIT* University of Pennsylvania Smell Identification Test, *DAT-SPECT* dopamine transporter single-photon emission computed tomography, ^a^UPSIT was not available in five subjects

The HC group consisted of 60 male volunteers of comparable aged 64.1 (SD 12.8) years, with no history of significant neurological or communication disorder. No group differences in age distribution were revealed between iRBD, PD and HC groups (ANOVA, *p* = 0.15).

### Speech examination

**Fig. 4:**
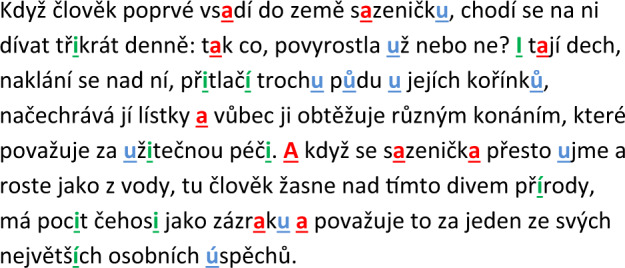
The text of the reading passage. Ten occurrences of each corner vowel used for the acoustic analysis are depicted by red (vowel /a/), green (vowel /i/) and blue (vowel /u/) colour.

The audio data were recorded in a quiet room with a low level of ambient noise using a head-mounted condenser microphone (Beyerdynamic Opus 55, Heilbronn, Germany) placed approximately 5 cm from the subject’s lips. The speech signals were sampled at 48 kHz with 16–bit resolution. The recordings were obtained during one session with a speech specialist (D.S., J.R., M.N. or T.T.) who conveyed instructions to the participants. Each participant completed a series of speaking tasks, including reading twice a standardised passage composed of 80 words as part of a longer protocol lasting about 20 minutes. A reading passage (Fig. 4), written by famous Czech writer Karel Capek, was chosen as it is a standardised speaking task representing a natural condition of connected speech that was further shown to possess the highest accuracy for discriminating iRBD from HC^11^. The second realization of the reading passage was utilized to minimize effect of patient’s natural ability to read aloud^42^. Neither signs of fatigue nor any changes in the quality of voice from the beginning to the end of the session were observed in any participant.

### Speech analyses

Acoustic measures were performed using the widely used speech-analysis software PRAAT (available at www.praat.org). Using PRAAT, both the combined wideband spectrographic display and the power spectral density were used to determine the first (F1) and the second (F2) formant frequencies in Hz. A total of 30 vowels per passage were studied, including 10 occurrences of /a/, 10 occurrences of /i/, and 10 occurrences of /u/ (Fig. 4). The formant frequencies of vowels /a/, /i/, and /u/ were extracted from a 30-ms segment at the temporal midpoint of the stable part of each vowel (to avoid the influence of vowels preceding or following); this method has been previously validated^43^ and proved to be reliable when applied to different languages^17,44,45^. The values of F1 and F2 frequencies were separately averaged for the individual corner vowel of each participant. The measurement of VSA and VAI were used. VSA is traditional and probably the most used articulatory-acoustic measure^46,47^, and can be easily calculated using the following formula:^48^

1 $$VSA = 0.5 \times \left( {\left[ {F2_{/u/} + F2_{/i/}} \right] \times \left[ {F1_{/u/} - F1_{/i/}} \right] - \left[ {F2_{/a/} + F2_{/u/}} \right] \times \left[ {F1_{/a/} - F1_{/u/}} \right] - \left[ {F2_{/a/} + F2_{/i/}} \right] \times \left[ {F1_{/a/} - F1_{/i/}} \right]} \right).$$

The measurement of VAI is another commonly used measure^46^ that was introduced by Roy et al ^49^. and can be expressed using the following formula^49^:

2 $$VAI = \left( {F2_{/i/} + F1_{/a/}} \right)/\left( {F1_{/i/} + F1_{/u/} + F2_{/u/} + F2_{/a/}} \right).$$

In addition, vowel duration was measured as the difference between the onset and offset of each vowel according to previously published methodology^50^. The final value used for statistical analysis was averaged across all 30 occurrences available.

Analysis of inter- and intra-judge reliability was not performed as the stability of methodology used for both healthy as well as dysarthric speech was thoroughly validated in previous studies^17,50^.

### Dopamine transporter imaging

In PD and RBD patients, we performed DAT-SPECT using the [123I]-2-b-carbomethoxy-3b-(4-iodophenyl)-N-(3-fluoropropyl) nortropane (DaTscan®, GE Healthcare) tracer according to European Association of Nuclear Medicine procedure guidelines^51^, using common acquisition and reconstruction parameters described in detail previously^52^. Automated semi-quantitative analysis was applied using the BasGan V2 software^53^, and specific binding ratios in both caudate nuclei and putamina relative to background binding were calculated; the lower value from both hemispheres was used for further analyses. Specific binding ratio values below the 95% reference prediction interval were considered abnormal.

### Statistical analyses

As the Kolmogorov-Smirnov test for independent samples showed that all acoustic variables were normally distributed, we used analysis of covariance (ANCOVA) with post hoc Bonferroni adjustment to assess group differences. Since there was wide variability in age among PD participants, age was considered as a covariate. The Pearson’s partial correlation analysis controlled for age was performed to test for significant relationships between the clinical and acoustic data. The level of significance was set to *p* < 0.05. All statistical analyses were performed in MATLAB (MathWorks, Natick, MA, USA).

### Reporting summary

Further information on research design is available in the Nature Research Reporting Summary linked to this article.

## Supplementary information

Reporting Summary

## Data Availability

Individual participant data that underlie the findings of this study are available upon request to the corresponding author by qualified researchers (i.e., affiliated to a respected university or research institution/hospital). The speech data are not publicly available due to their contain of information that could compromise the privacy of study participants.
