# Supplementary material for: Articulatory undershoot of vowels in isolated REM sleep behavior disorder and early Parkinson’s disease
Source: NPJ Parkinsons Dis. 2022 Oct 20;8:137. doi: 10.1038/s41531-022-00407-7 (PMC9584921; doi:10.1038/s41531-022-00407-7)
Supplement: Supplementary file 1 — Reporting Summary [file 41531_2022_407_MOESM1_ESM.pdf]

nature portfolio

Corresponding author(s): Tereza Tykalova

Last updated by author(s): Aug 19, 2022

Reporting Summary

Nature Portfolio wishes to improve the reproducibility of the work that we publish. This form provides structure for consistency and transparency in reporting. For further information on Nature Portfolio policies, see our Editorial Policies and the Editorial Policy Checklist.

Statistics

For all statistical analyses, confirm that the following items are present in the figure legend, table legend, main text, or Methods section.

n/a	Confirmed	
☐	☒	The exact sample size (*n*) for each experimental group/condition, given as a discrete number and unit of measurement
☐	☒	A statement on whether measurements were taken from distinct samples or whether the same sample was measured repeatedly
☐	☒	The statistical test(s) used AND whether they are one- or two-sided<br>*Only common tests should be described solely by name; describe more complex techniques in the Methods section.*
☐	☒	A description of all covariates tested
☐	☒	A description of any assumptions or corrections, such as tests of normality and adjustment for multiple comparisons
☐	☒	A full description of the statistical parameters including central tendency (e.g. means) or other basic estimates (e.g. regression coefficient) AND variation (e.g. standard deviation) or associated estimates of uncertainty (e.g. confidence intervals)
☐	☒	For null hypothesis testing, the test statistic (e.g. *F*, *t*, *r*) with confidence intervals, effect sizes, degrees of freedom and *P* value noted<br>*Give P values as exact values whenever suitable.*
☒	☐	For Bayesian analysis, information on the choice of priors and Markov chain Monte Carlo settings
☒	☐	For hierarchical and complex designs, identification of the appropriate level for tests and full reporting of outcomes
☐	☒	Estimates of effect sizes (e.g. Cohen's *d*, Pearson's *r*), indicating how they were calculated

Our web collection on statistics for biologists contains articles on many of the points above.

Software and code

Policy information about availability of computer code

Data collection	No software was used.
Data analysis	All analyses were performed in MATLAB® (MathWorks, Natick, MA).

For manuscripts utilizing custom algorithms or software that are central to the research but not yet described in published literature, software must be made available to editors and reviewers. We strongly encourage code deposition in a community repository (e.g. GitHub). See the Nature Portfolio guidelines for submitting code & software for further information.

Data

Policy information about availability of data

All manuscripts must include a data availability statement. This statement should provide the following information, where applicable:

- Accession codes, unique identifiers, or web links for publicly available datasets
- A description of any restrictions on data availability
- For clinical datasets or third party data, please ensure that the statement adheres to our policy

Individual participant data that underlie the findings of this study are available upon reasonable request from the corresponding author. The speech data are not publicly available due to their contain of information that could compromise the privacy of study participants.

Human research participants

Policy information about studies involving human research participants and Sex and Gender in Research.

Reporting on sex and gender	We enrolled exclusively male participants primarily because of the strong predominance of male subjects within iRBD patients. We also admit this within the limitation section. A total of 180 male Czech participants, including a separate group of patients with Isolated rapid eye movement sleep disorder (n=60), patients with Parkinson's disease PD (n=60), and healthy control participants (n=60), were recruited.
Population characteristics	A total of 180 male Czech participants, including a separate group of patients with Isolated rapid eye movement sleep disorder (n=60), patients with Parkinson's disease PD (n=60), and healthy control participants (n=60), were recruited. The iRBD group consisted of 60 male patients aged 65.6 (SD 7.1) years diagnosed according to the third edition of the International Classification of Sleep Disorders. The PD group consisted of 60 untreated drug-naïve male patients aged 61.8 (SD 11.6) years, fulfilling the Movement Disorder Society clinical diagnostic criteria for PD. The HC group consisted of 60 male volunteers of comparable aged 64.1 (SD 12.8) years, with no history of significant neurological or communication disorder
Recruitment	The PD and iRBD patients were recruited at General University Hospital in Prague. The control subjects were recruited from the general community through advertisements. No selection bias was present.
Ethics oversight	The study was approved by the Ethics Committee of the General University Hospital in Prague, Czech Republic and have therefore been performed in accordance with the ethical standards laid down in the 1964 Declaration of Helsinki and its later amendments. All participants provided written, informed consent to the neurological examination and recording procedure.

Note that full information on the approval of the study protocol must also be provided in the manuscript.

Field-specific reporting

Please select the one below that is the best fit for your research. If you are not sure, read the appropriate sections before making your selection.

☐ Life sciences ☒ Behavioural & social sciences ☐ Ecological, evolutionary & environmental sciences

For a reference copy of the document with all sections, see nature.com/documents/nr-reporting-summary-flat.pdf

Behavioural & social sciences study design

All studies must disclose on these points even when the disclosure is negative.

Study description	Quantitative cross-sectional study.
Research sample	A total of 180 concecutive male Czech participants, including a separate group of patients with Isolated rapid eye movement sleep disorder (n=60), patients with Parkinson's disease PD (n=60), and healthy control participants (n=60), were recruited.
Sampling strategy	An ad-hoc power analysis based on one-way analysis of covariance with one factor (GROUP) and one covariate (AGE) indicated a recommended minimum overall sample size of 64 for 3 groups (i.e., a minimum sample size of 22 per one group), given expected large effect size (Cohen's f of 0.4) with the error probability α set at 0.05 and a false negative rate β set at 0.2 (i.e., power of 0.8). We included a sample size of 180 subjects for 3 groups (i.e., sample size of 60 per one group).
Data collection	Speech recordings were performed in a quiet room with a low ambient noise level using a head-mounted condenser microphone (Beyerdynamic Opus 55, Heilbronn, Germany) placed approximately 5 cm from the subject's mouth. Speech signals were sampled at 48 kHz with 16-bit resolution. Each subject was recorded during a single session with a speech specialist. Researcher was blind to the study hypothesis during data collection.
Timing	From 2015 to 2021
Data exclusions	No data were excluded from the analyses.
Non-participation	No participants fullfilling inclusion/exlusion criteria stated within manuscript dropped from the analyses.
Randomization	A movement disorders specialist established the clinical diagnoses of all patients. The iRBD group consisted of 60 male patients diagnosed according to the third edition of the International Classification of Sleep Disorders. The PD group consisted of 60 untreated drug-naïve male patients fulfilling the Movement Disorder Society clinical diagnostic criteria for PD.

Reporting for specific materials, systems and methods

We require information from authors about some types of materials, experimental systems and methods used in many studies. Here, indicate whether each material, system or method listed is relevant to your study. If you are not sure if a list item applies to your research, read the appropriate section before selecting a response.

Materials & experimental systems

n/a	Involved in the study
☒	☐ Antibodies
☒	☐ Eukaryotic cell lines
☒	☐ Palaeontology and archaeology
☒	☐ Animals and other organisms
☒	☐ Clinical data
☒	☐ Dual use research of concern

Methods

n/a	Involved in the study
☒	☐ ChIP-seq
☒	☐ Flow cytometry
☒	☐ MRI-based neuroimaging
